# Resistance to Beta-cypermethrin, Azadirachtin, and Matrine, and Biochemical Characterization of Field Populations of *Oedaleus asiaticus* (Bey-Bienko) in Inner Mongolia, Northern China

**DOI:** 10.1093/jisesa/ieac063

**Published:** 2022-11-14

**Authors:** Shujing Gao, Yao Tan, Haibin Han, Na Guo, Haiyan Gao, Linbo Xu, Kejian Lin

**Affiliations:** Institute of Grassland Research, Chinese Academy of Agricultural Science, Hohhot 010010, Inner Mongolia, China; Research Center for Grassland Entomology, Inner Mongolia Agricultural University, Hohhot 010020, China; Institute of Grassland Research, Chinese Academy of Agricultural Science, Hohhot 010010, Inner Mongolia, China; Institute of Grassland Research, Chinese Academy of Agricultural Science, Hohhot 010010, Inner Mongolia, China; Institute of Grassland Research, Chinese Academy of Agricultural Science, Hohhot 010010, Inner Mongolia, China; Institute of Grassland Research, Chinese Academy of Agricultural Science, Hohhot 010010, Inner Mongolia, China; Institute of Grassland Research, Chinese Academy of Agricultural Science, Hohhot 010010, Inner Mongolia, China

**Keywords:** resistance monitoring, *Oedaleus asiaticus*, synergism, detoxification enzyme, insecticide

## Abstract

*Oedaleus asiaticus* (Bey-Bienko) is an economically devastating locust species found in grassland and pastoral areas of the Inner Mongolia region of northern China. In this study, resistance to three frequently used insecticides (beta-cypermethrin, matrine, and azadirachtin) was investigated in six field populations of *O. asiaticus* using the leaf-dip bioassay method. The inhibitory effects of synergists and the activities of detoxification enzyme activities in the different populations were determined to explore potential biochemical resistance mechanisms. The results showed that the field populations SB (resistance ratio [RR] = 7.85), ZB (RR = 5.64), and DB (RR = 6.75) had developed low levels of resistance to beta-cypermethrin compared with a susceptible control strain. Both the SB (RR = 5.92) and XC (RR = 6.38) populations had also developed low levels of resistance against matrine, with the other populations remaining susceptible to both beta-cypermethrin and matrine. All field populations were susceptible to azadirachtin. Synergism analysis showed that triphenyl phosphate (TPP) and diethyl-maleate (DEM) increased the toxicity of beta-cypermethrin significantly in the SB population, while the synergistic effects of TPP, piperonyl butoxide (PBO), and DEM on the toxicity of matrine were higher in SB (SR 3.86, 4.18, and 3.07, respectively) than in SS (SR 2.24, 2.86, and 2.29, respectively), but no synergistic effects of TPP, PBO, and DEM on azadirachtin were found. Biochemical assays showed that the activities of carboxylesterases (CarEs) and glutathione-*S*-transferases (GSTs) were significantly raised in all field populations of *O. asiaticus*, with a significant positive correlation observed between beta-cypermethrin resistance and CarE activity. The activities of cytochrome P450 monooxygenases (P450) and multi-function oxidases (MFO) were elevated in all six field populations, and P450 activity displayed strong positive correlations with the three insecticides. Our findings suggest that resistance to beta-cypermethrin in *O. asiaticus* may be mainly attributed to elevated CarE and GST activities, while P450 plays an important role in metabolizing matrine and azadirachtin. Our study provides insights that will help improve insecticide resistance management strategies.

The band-winged locust, *Oedaleus asiaticus* (Orthoptera, Acrididae), is an economically important pest that is widely distributed throughout the northern Asian grasslands (Chen and Kang 2000), and is particularly abundant on the steppes of the Inner Mongolian, Gansu, Hebei, Shanxi, Shandong region of northern China, and mainly in Mongolia, Russia, and other regions abroad. (Li and Ma 1990, Zhou et al. 2012). This locust species prefers to live on overgrazed steppes and mainly feeds on gramineaceous plants such as *Stipa krylovii*, *Stipa grandis*, *Neurolepidium chinense*, and *Cleistogenes squarrosa* (Huang et al. 2016, 2017). In Inner Mongolia, *O. asiaticus* is characterized by one generation annually and occasionally exhibits migratory behavior (Cease et al. 2010). This pest causes great damage to the vegetation on grasslands and they have been suggested to serve as indicators of habitat deterioration in typical steppe zone of Inner Mongolia (Kang and Chen 1995, Liu and Guo 2004). With the gradual exacerbation of climate change and heavy livestock pressure, *O. asiaticus* has become increasingly destructive, threatening agriculture, and animal husbandry in northern China, especially in Inner Mongolia (Liu et al. 2013, Zhang et al. 2014).

Although diverse strategies have been developed to control outbreaks of locusts and grasshoppers, the use of broad-spectrum insecticides remains the most effective control tactic (Cease et al. 2012, Zhang et al. 2019). Over the last 20 years, conventional insecticides, including pyrethroids and neonicotinoids, have been widely recommended against locusts in steppe regions (Mao et al. 2021). However, the frequent use of insecticides has led to the development of resistance and consequent loss of efficacy (Li et al. 2014, Cao et al. 2017). Therefore, determining the susceptibility of field populations of *O. asiaticus* to common insecticides and elucidating its resistance mechanisms are important for the implementation of suitable management strategies against this key pest.

In recent years, *O. asiaticus* has developed resistance to numerous insecticides, including beta-cypermethrin and deltamethrin (Dong et al. 2016, Cao et al. 2017). Beta-cypermethrin is frequently used to control *O. asiaticus* on the Inner Mongolian steppe, where populations have a low level of resistance (Dong et al. 2016). Two botanical insecticides, azadirachtin and matrine, also play an important role in locust control, and their long-term usage has thus far raised little concern about the development of resistance (Zhang et al. 2018). Previous studies have observed that pyrethroid resistance is associated with elevated levels of detoxification enzymes such as cytochrome P450 monooxygenases (P450s) (Sonoda 2010, Silva et al. 2015, Zibaee et al. 2018), multi-function oxidases (MFOs) (Chen et al. 2019), carboxylesterases (CarEs) (Xu et al. 2021), and glutathione-*S*-transferases (GSTs) (Vontas et al. 2001; Yang et al. 2004) in a wide range of insect species. The use of botanical pesticides has led to adaptation in detoxification enzymes, possibly resulting in resistance to a variety of chemicals (Tan et al. 2019). Giraudo et al. (2015) found that overexpression of P450 genes was induced by azadirachtin and showed the important role of the CYP3 enzymes in the metabolism of botanical pesticides. Shu et al. (2021) speculated that the up-regulation of P450 and GST enzymes may be involved in the degradation of azadirachtin in *Spodoptera frugiperda* larvae.

In the present study, we monitored the resistance levels of *O. asiaticus* against beta-cypermethrin, azadirachtin, and matrine. Populations collected from six regions of the Inner Mongolian steppe were exposed to these three insecticides. In addition, to investigate the metabolic mechanisms that are involved in resistance, three synergist compounds, piperonyl butoxide (PBO), diethyl-maleate (DEM), and triphenyl phosphate (TPP), were used to determine the effects of the detoxifying enzyme activities on the sensitivity of these field populations of *O. asiaticus* towards these insecticides. Detoxification enzyme activities were measured in the different field populations to determine underlying biochemical resistance mechanisms. Our results provide a framework for an integrated management strategy using commonly used insecticides against the locust on the Inner Mongolian steppes.

## Materials and Methods

### Insects

**Fig. 1.**
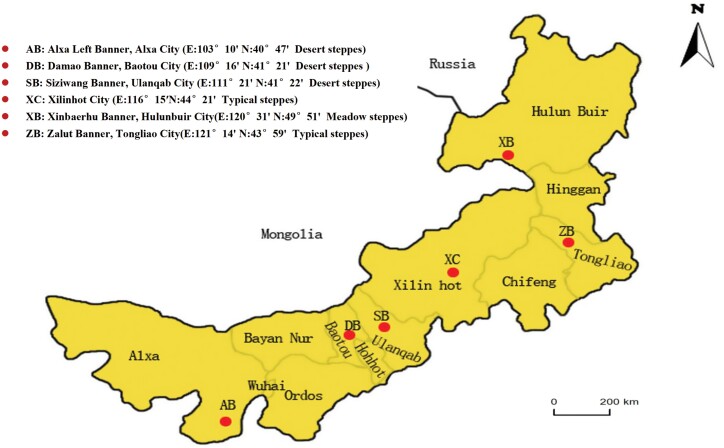
Collection locations of the six *O. asiaticus* populations tested.

A susceptible strain (SS) of *O. asiaticus* was collected in June 2018 from the Daqing Mountain grassland area (111° 38ʹ16.2″ E, 41° 21ʹ12.6″ N) in Hohhot, Inner Mongolia, China. Daqing Mountain area is a national nature reserve, where pesticides have not been used and *O. asiaticus* resistance to insecticides has never been reported. The susceptible strain has been reared in the laboratory without exposure to insecticides. Geographical populations of *O. asiaticus* were collected from six sampling areas in the Inner Mongolian steppe region in June 2020. These areas included typical steppe regions in Xilinhot City (XC) and Zarut Banner (ZB), desert steppes in Siziwang Banner (SB), Alxa Left Banner (AB), and Damao Banner (DB), and meadow steppes in Xinbaerhu Banner (XB) (Fig. 1). All locust strains were reared on wheat seedlings in isolated cages with mesh protection and maintained in the laboratory without any exposure to insecticides at 26 ± 1°C, 60 ± 5% relative humidity (RH), and a photoperiod of 14:10 (L:D) h. The wheat variety used was Changfeng 2112, and the seeds were purchased from Shaanxi Xichangfeng Seed Industry Co., Ltd. (China). The different populations were kept separately in the laboratory and the third-instar nymphs were immediately processed for bioassays or frozen in liquid nitrogen and stored at −80°C for biochemical determinations.

### Reagents

The active insecticide ingredients beta-cypermethrin (96%), azadirachtin (30%), and matrine (98%) were provided by Beijing Qingyuanbao Biological Technology Co., Ltd. (Beijing, China). Piperonyl butoxide (PBO; 98%) and diethyl maleate (DEM; 97%) were purchased from Sigma-Aldrich Co. (St Louis, MO). Triphenyl phosphate (TPP), α-naphthol, α-naphthyl acetate (α-NA), β-naphthyl acetate (β-NA), and fast blue B salt (O-dianisidine, tetrazotized) were acquired from Shanghai No.1 Chemical Reagent Company (China); bovine serum albumin (BSA) was from Bio-Rad (Hercules, CA); sodium dodecyl sulfate (SDS) was bought from Sigma Chemical Co.; Coomassie brilliant blue G-250, sucrose, and Triton X-100 were bought from Amresco Co. (Solon, OH); acrylamide (Acr) and disodium ethylenediaminetetraacetate (EDTA-Na_2_) were obtained from Solarbio (Beijing, China). In addition, 1,2-dichloro-4-nitrobenzene (DCNB), acetylthiocholine iodide (ATChI), and catechol,1-chloro-2,4-dinitrobenzene (CDNB) were bought from Sigma Chemical Co.). Reduced glutathione, p-nitrophenol, p-nitroanisole, and coenzyme NADPH were obtained from Shanghai Dingguo Biotech Development Co. (Shanghai, China). N,N,Nʹ,Nʹ-tetramethyl ethylenediamine (TEMED) and other reagents were purchased from commercial suppliers in China.

### Bioassay and Synergism Determination

The susceptibility of *O. asiaticus* to insecticides was determined using the leaf-dip bioassay method as described by Shelton (1993) with minor modifications. Stock solutions of each of the three insecticides were prepared in acetone and seven dilutions, causing 10–90% mortality, were prepared. Fresh wheat seedling bundles were cut into small 3-cm-diameter pieces. Each bundle was dipped in a solution of the diluted insecticide or acetone as a control for 10 s, and allowed to air-dry at room temperature. The bundles were placed individually inside a plastic feeding box (20 cm × 15 cm × 6 cm). Third-instar nymphs of *O. asiaticus* were selected and used for the bioassay. Each treatment was replicated three times with each replication containing 20 test locusts. The control nymphs were treated with a 1% acetone solution. All third-instar nymphs were kept at 26 ± 1°C, 60 ± 5% RH, and a photoperiod of 14:10 (L:D) h, and the mortality was recorded after 48 h exposure. The individuals were considered dead if they failed to move or twitch slightly when touched with a fine brush.

To study which detoxifying enzymes might play critical roles in the mechanism of insecticide resistance, bioassays were performed in the absence or presence of the synergists TPP, PBO, and DEM. The mixing ratio was 3:1 (synergist/insecticide) according to He et al. (2009). The field population with the highest resistance level to the three insecticides was used for synergism determination.

### Detoxification Enzyme Activity Determination

#### Enzyme Extract Preparation

Thorax samples of third-instar nymphs from each geographical population were dissected on ice and homogenized in phosphate-buffered saline (PBS) (0.1 mol L^−1^, pH 7.5) containing 0.3% (v:v) Triton X-100. The homogenates were centrifuged at 10,000 g for 10 min at 4°C. The supernatants were transferred to new centrifuge tubes and centrifuged again at 15,000 × *g* for 20 min at 4°C. The supernatants were then used for the immediate measurement of protein concentrations and enzyme activities.

#### Carboxylesterase (CarE) Activity

Carboxylesterase (CarE) activity was assayed using the method described by Zhu and He (2000). Enzyme-containing solutions were added to microplate wells with the substrates α-NA (3 × 10^−4^ mol L^−1^) and physostigmine (1 × 10^−4^ mol L^−1^) and incubated at 37°C for 10 min. The chromogenic agent included 5% SDS and 1% fast blue B salt (v/v = 5:2). OD values at 600 nm were measured in a microplate reader (Synergy H4, Burton Co.).

#### Glutathione S-transferase Activity

The activity of glutathione S-transferase (GST) was determined according to the method of Oppenoorth et al. (1979) with minor modifications. The enzyme-containing solutions were added to microplate wells with the substrates CDNB (0.6 mmol L^−1^) and GSH (6 mol L^−1^), and then incubated at 37°C for 20 min. Absorbances at 340 nm were read at 30-second intervals for 5 min.

#### Multifunction Oxidase Activity

Multifunction oxidase (MFO) activity was measured using a modification of the method of Shang and Soderlund (1984). The enzyme solution was added to microplate wells with the substrates nitroanisole (0.05 mol L^−1^ in acetone) and NADPH (9.6 mmol L^−1^) and incubated for 30 min at 37°C. The reaction was terminated with 1 mol L^−1^ hydrochloric acid, and extracted with chloroform and NaOH (0.5 mol L^−1^). The OD values were measured at 405 nm using a microplate reader.

#### Cytochrome P450 Monooxygenase (P450) Activity

The activity of P450 was evaluated by measuring p-nitroanisole (PNOD) activities according to the method of Chang and Hodgson (1975) with minor modifications. The detection system contained 100 µL of enzyme solution, 25 mM p-nitroanisole, and 100 mM potassium phosphate buffer (pH 7.2). After the addition of 100 mM D-glucose-6-phosphate sodium salt and 100 U/mL glucose-6-phosphate dehydrogenase, the reaction was initiated by the addition of 5 mM beta-nicotinamide adenine dinucleotide phosphate. After incubation for 10 min at 30°C, the reaction was stopped by adding acetone containing 2 mM glycine and 2 U/mL sodium hydroxide. Absorbances at 405 nm were measured in the microplate reader and the amount of product formed was calculated from the p-nitrophenol standard curve. CarE, GST, P450, and MFO activities were measured in all field populations and were compared to the susceptible strain (SS).

For all the experiments of detoxification enzyme activity determination, each geographical population was as one treatment group, there are a total of 7 geographical populations or 7 treatment groups including susceptible strain (SS) (SS, AB, DB, SB, XC, XB, and ZB). The experiments were performed three determinations with three biological replicates per geographical sample (treatment group).

### Protein Quantification

Protein concentration was determined by the method of Bradford (1976), using bovine serum albumin as the standard.

### Data Analysis

Mortality data were corrected using Abbott’s formula (1987) and analyzed by probit analysis using POLO-Plus (Robertson et al. 2007). Differences were considered significant if the 95% confidence limits of the two LC_50_ values did not overlap. Differences in enzyme activities were analyzed using one-way analysis of variance (ANOVA) with Tukey’s test for means separation and multiple linear regression was used to analyze the correlation between resistance ratio to insecticide and the enzymatic activity of the population using SPSS 18.0 software. Enzymatic activities were expressed as mean ± SE (mol μg^−1^min^−1^ protein for CarEs, GSTs, P450s, and MFOs). Softmax Pro 6.1 was used to record and analyze the protein data.

Resistance levels were classified based on the resistance ratio (RR) using LC_50_ values compared with susceptible populations. Resistance levels were classified as follows: susceptible (RR = 1–5 fold), low resistance (RR = 5–10 fold), medium resistance (RR = 10–40 fold), or high resistance (RR = 40–160 fold) (Kim et al. 1999).

## Results

### Monitoring of Resistance of *O. asiaticus* to Insecticides

The LC_50_ values of the SS population with beta-cypermethrin, matrine, and azadirachtin were 0.21 µg L^–1^, 0.43 µg L^–1^, and 0.54 µg L^–1^, respectively (Table 1). This showed that the SS population of *O. asiaticus* was more susceptible to beta-cypermethrin, matrine, and azadirachtin than several of the field collected populations.

**Table 1.** Resistance ratios towards beta-cypermethrin, matrine, and azadirachtin of field populations of *O. asiaticus* from Inner Mongolia compared to a susceptible strain (SS)

Insecticides	Population	χ2^a^	Slope ± SE	LC_50_ (µg L^−1^)	95% CL	RR^b^ (R/S)
Beta-cypermethrin	SS	2.36^*^	2.30 ± 0.51	0.21	0.17–0.30	1.00
	AB	1.67^*^	1.83 ± 0.39	0.77	0.46–1.13	3.68
	DB	4.55^*^	2.67 ± 0.41	1.41	0.82–1.94	6.72
	SB	2.14^*^	3.13 ± 0.58	1.65	0.73–2.15	7.85
	XC	0.97^*^	1.16 ± 0.36	0.78	0.46–1.14	3.74
	XB	2.18^*^	2.34 ± 0.42	0.99	0.49–1.36	4.76
	ZB	1.45^*^	1.68 ± 0.33	1.13	0.67–1.45	5.64
Matrine	SS	0.83^*^	1.63 ± 0.38	0.43	0.42–0.65	1.00
	AB	1.97^*^	1.97 ± 0.41	1.71	1.32–3.75	3.98
	DB	2.34^*^	1.82 ± 0.36	2.05	1.75–4.25	4.76
	SB	1.20^*^	2.63 ± 0.47	2.55	2.30–4.16	5.92
	XC	3.19^*^	2.06 ± 0.41	2.74	2.43–4.70	6.38
	XB	4.15^*^	1.89 ± 0.39	0.92	0.83–2.27	2.13
	ZB	1.74^*^	2.24 ± 0.41	1.49	1.31–3.75	3.46
Azadirachtin	SS	2.13^*^	3.34 ± 0.64	0.54	0.31–0.59	1.00
	AB	3.62^*^	3.22 ± 0.61	1.25	0.62–1.74	2.32
	DB	2.27^*^	3.01 ± 0.54	1.31	0.67–1.59	2.41
	SB	5.01^*^	2.15 ± 0.46	1.39	0.73–1.66	2.57
	XC	0.92^*^	1.79 ± 0.29	1.05	0.29–1.82	1.95
	XB	1.67^*^	1.68 ± 0.28	0.95	0.35–1.57	1.76
	ZB	2.54^*^	2.10 ± 0.31	0.85	0.32–1.62	1.56

^*^Meant pass the χ^2^ test.

^b^Pearson chi-square, goodness-of-fit test.

^c^RR: resistance ratio = LC_50_ of the field strain/LC_50_ of the susceptible strain.

The LC_50_ values of field collected populations ranged from 0.773 to 1.648 µg L^ − 1^ for beta-cypermethrin, and the resistance ratios (RRs) ranged from 3.68 to 7.85. Table 1 indicates that the SB, ZB, and DB populations had developed low resistance to beta-cypermethrin. The SB and XC populations had developed low resistance towards matrine, with 5.92- and 6.38-fold higher LC_50_ values, respectively, while the other populations remained susceptible (Table 1). The resistance levels in the field populations of *O. asiaticus* exhibited low LC_50_ values for azadirachtin, with RRs ranging from 1.56 to 2.57, suggesting that the six populations remained susceptible to azadirachtin (Table 1).

### Synergism Effects

The SB population, with a trend for being more resistant to beta-cypermethrin, matrine, and azadirachtinhad than the other field populations, was chosen to analyze the potential biochemical mechanism of resistance in *O. asiaticus*. Both TPP and DEM increased the beta-cypermethrin toxicity for the SB population, with synergistic ratios (SRs) of 5.29 and 4.92, respectively, significantly higher than those in the SS (SRs of 2.05 and 2.67, respectively). No synergistic effects of PBO were observed in both the SS and the SB populations (SR < 2.0). The synergistic effects of TPP, PBO, and DEM on the toxicity of matrine were higher in the SB (SRs 3.86, 4.18, and 3.07, respectively) than in the SS (SRs 2.24, 2.86, and 2.29, respectively). However, no synergistic effects of TPP, PBO, and DEM on azadirachtin were found in the SS or SB populations (Table 2).

**Table 2.** Synergism with beta-cypermethrin, matrine, and azadirachtin between SS and SB strains

Insecticides	population	χ^2^^a^	Slope ± SE	LC_50_ (µg L^−1^)	95% CL	SR^b^
Beta-cypermethrin	SS	2.36^*^	2.30 ± 0.51	0.21	0.17–0.30	–
	SB	2.14^*^	3.13 ± 0.58	1.65	0.73–2.15	–
Beta-cypermethrin + TPP	SS	1.95^*^	1.67 ± 0.36	0.10	0.09–0.18	2.05
	SB	3.01^*^	2.81 ± 0.42	0.31	0.25–0.66	5.29
Beta-cypermethrin + PBO	SS	0.94^*^	1.17 ± 0.27	0.20	0.17–0.28	1.02
	SB	1.64^*^	1.85 ± 0.42	0.83	0.59–1.28	1.98
Beta-cypermethrin + DEM	SS	1.26^*^	1.54 ± 0.32	0.08	0.07–0.15	2.67
	SB	0.92^*^	2.15 ± 0.41	0.33	0.26–0.59	4.92
Matrine	SS	0.83^*^	1.63 ± 0.38	0.43	0.42–0.65	–
	SB	1.20^*^	2.63 ± 0.47	2.55	2.30–4.16	–
Matrine + TPP	SS	2.67^*^	0.63 ± 0.21	0.19	0.16–0.27	2.24
	SB	3.55^*^	1.14 ± 0.32	1.14	0.94–1.41	3.86
Matrine + PBO	SS	3.47^*^	1.89 ± 0.54	0.15	0.13–0.19	2.86
	SB	6.24^*^	4.67 ± 0.36	0.61	0.40–0.71	4.18
Matrine + DEM	SS	2.47^*^	1.54 ± 0.39	0.18	0.17–0.20	2.29
	SB	1.69^*^	0.72 ± 0.24	0.83	0.58–0.93	3.07
Azadirachtin	SS	2.13^*^	3.34 ± 0.64	0.54	0.31–0.59	–
	SB	2.89^*^	0.88 ± 0.24	1.38	0.73–1.66	–
Azadirachtin + TPP	SS	3.44^*^	1.96 ± 0.41	0.25	0.19–0.35	2.16
	SB	3.53^*^	2.11 ± 0.38	0.42	0.28–0.56	3.28
Azadirachtin + PBO	SS	1.24^*^	2.99 ± 0.57	0.26	0.16–0.41	2.08
	SB	3.14^*^	1.36 ± 0.32	0.33	0.21–0.49	4.19
Azadirachtin + DEM	SS	3.68^*^	2.94 ± 0.39	0.24	0.12–0.38	2.25
	SB	2.86^*^	1.71 ± 0.51	0.52	0.37–0.63	2.65

^*^Meant pass the χ^2^ test.

^a^Pearson chi-square, goodness-of-fit test.

^b^SR: synergistic ratio = LC_50_ of insecticides/LC_50_ of (synergist + insecticides).

### Detoxification Enzyme Activities

**Fig. 2.**
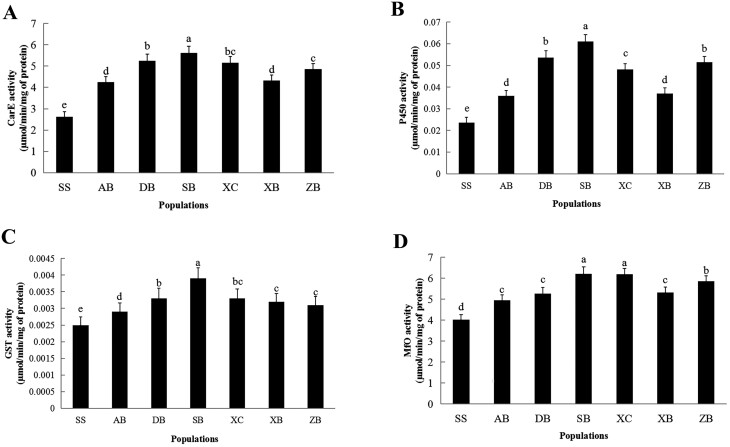
Activities of detoxifying enzymes in *O. asiaticus* (locust) populations from Inner Mongolia. The activities of Carboxylesterase (CarEs) (A), Cytochrome P450 monooxygenase (B), Glutathione *S*-transferase (C), and Mixed-function oxidase (D) were determined for six field populations and a susceptible strain (SS) of *O. asiaticus*. The results are means ± SE, bars marked with different letters are significantly different at *P < *0.05 by ANOVA with Bonferroni multiple comparison test [*F*_(6,56)_ = 191.62, *P* < 0.0001 (CarE); *F*_(6,56)_ = 225.94, *P* < 0.0001 (P450); *F*_(6,56)_ = 127.65, *P* = 0.0001 (GSTs); *F*_(6,56)_ = 5.97, *P* = 0.0027].

Activities for CarE ranged from 4.26 to 5.62 in the field populations compared with 2.63 μmol/min/mg protein in the SS (Fig. 2A) (*F *= 191.62; df = 6, 56; *P* < 0.0001) These results indicated that the CarE activity was significantly higher in the six field populations than in the susceptible population (*P* < 0.05); the CarE activity ratios of the SB, DB, XC, ZB, XB, and AB populations were 2.14, 2.00, 1.96, 1.85, 1.65, and 1.62-fold, respectively. Correlation analysis showed a significant positive correlation between the RRs to beta-cypermethrin and CarE activity in different field populations (Table 3).

**Table 3.** Correlation coefficients (*r*) between insecticide toxicities and enzyme activities in *O. asiaticus*

Insecticides	CarEs	GST	MFO	P450
Beta-cypermethrin	0.932^*^ (0.016)	0.757 (0.212)	0.416 (0.562)	0.842 (0.324)
Matrine	0.556 (0.445)	0.463 (0.452)	0.327 (0.614)	0.905 (0.486)
Azadirachtin	0.572 (0.383)	0.379 (0.687)	0.285 (0.796)	0.917 (0.227)

The figures in parentheses indicate the probability rejecting null hypothesis that *r *= 0.

^*^Represents significant correlation at *P > *0.01.

The activities of cytochrome P450 monooxygenases (P450s) were elevated in all six field populations, ranging from 0.04 to 0.06 μmol/min/mg of protein compared with 0.02 μmol/min/mg of protein in the SS population (*F *= 225.94; df = 6, 56; *P* < 0.0001), with the SB population showing the highest activity ratio of 2.54-fold (Fig. 2B). Correlation coefficients between the RRs for the three insecticides and P450 activities were not significant (Table 3).

GST activity ranged from 2.9 to 3.9 nmol/min/mg protein in the six field populations compared with 2.5 nmol/min/mg protein in the SS (Fig. 2C) (*F *= 127.65; df = 6, 56; *P* = 0.0001). The activity ratios were 1.16–1.56-fold compared to the SS population (*P < *0.05). The correlation coefficients between GST and the RRs for beta-cypermethrin, matrine, and azadirachtin were not significant (Table 3).

MFO activities were elevated in all six field populations, ranging from 4.96 to 6.21 μmol/min/mg protein compared with 4.02 μmol/min/mg protein in the SS population (*F *= 5.97; df* *= 6, 56; *P* = 0.0027) (Fig. 2D). The highest activity ratio was 1.54-fold in the SB population. The correlation coefficients between MFO activity and RRs for beta-cypermethrin, matrine, and azadirachtin were not significant (Table 3).

## Discussion

Development of resistance in locusts to various groups of insecticides is a major concern for sustainable management of this pest. Our results showed that field populations of locusts from different regions of the Inner Mongalian steppes had varying levels of resistance to three insecticides. Compared with the SS population, resistance levels of six field populations to beta-cypermethrin were still at a low level. The SB and XC populations had low-level resistance to matrine with resistance ratios (RRs) of 5.92 and 6.38, respectively, which is likely associated with the frequent use of matrine for locust control in recent years (Bilal et al. 2018). In the past 10 years, beta-cypermethrin has been used for significant locust outbreaks, while matrine is used more frequently for routine control (Ma et al. 2018). All field populations were susceptible to azadirachtin.

Increased levels of detoxification enzyme activities can play an important role in the early stages of resistance development (Sonoda 2010, Lira et al. 2020). The synergistic inhibition of detoxification enzymes may increase the sensitivity of locusts to the tested insecticides. Based on our results, TPP and DEM had a synergistic effect on beta-cypermethrin resistance with activity ratios of 5.29 and 4.92, respectively, for the SB population, indicating that both CarE and GST play important roles in the resistance of *O. asiaticus* to beta-cypermethrin. Results also showed that PBO and TPP had a greater synergistic effects with matrine and azadirachtin, suggesting that CarE and MFO are closely associated with the resistance of *O. asiaticus* to these botanical insecticides. Many secondary metabolites that are produced in plants, including alkaloids and terpenes, have strong insecticidal activity that has been exploited for developing botanical insecticides. In response, insects have evolved multiple detoxification strategies for metabolizing plant toxins (Tan et al. 2019).

In the present study, elevated CarE and GST activities appeared to be associated with the resistance of *O. asiaticus* to beta-cypermethrin. CarE activity was significantly elevated in all field populations and a significant positive correlation was shown between beta-cypermethrin resistance and CarEs activity in *O. asiaticus*. Similarly, an association between elevated CarE levels and resistance to beta-cypermethrin has been reported in the house fly, *Musca domestica* (Zhang et al. 2007, 2010). Elevated CarE activities have been implicated as a biochemical resistance mechanism in many insect species due to their ability to hydrolyze the ester bond of insecticides (Hemingway 2000, Wu et al. 2011). Increased GST activity was found in the six field populations, but statistical analysis failed to establish any significant correlation between GST activity and beta-cypermethrin resistance. Previous studies have demonstrated that GST plays an important role in pyrethroid resistance in many insect species (Vontas et al. 2001, Enayati and Haghi 2007, Achaleke et al. 2009, Cao et al. 2017). Therefore, the enhanced GST activity in these populations may be due to resistance developed against other insecticides that had been previously applied. P450 activity was significantly increased in all six field populations and the activity ratios were between 1.57 and 2.54-fold higher than in the SS. Correlation coefficients between resistance to botanical insecticides (matrine and azadirachtin) and P450 activity were 0.91 and 0.92 for matrine and azadirachtin, respectively, suggesting that these insecticides might trigger P450-associated detoxification mechanisms. Tan (2019) also reported the important role of P450s induced by azadirachtin in xenobiotic metabolism.

In conclusion, the populations of *O. asiaticus* from the AB, DB, SB, XC, XB, and ZB regions of Inner Mongolia have developed different degrees of resistance to the three selected insecticides. The high selection pressure with beta-cypermethrin and matrine insecticides in the field likely contributed to the resistance emergence of *O. asiaticus* populations, which are resistant to more than one insecticide. Potential biochemical resistance mechanisms in *O. asiaticus* against these insecticides were explored using synergists. Our study showed that the activities of detoxification enzymes of *O. asiaticus* were associated with resistance levels. The results suggested that the variations in *O. asiaticus* susceptibility to beta-cypermethrin might be attributed to increased activities of CarE and GST, while CarE and MFO might play important roles in metabolizing the plant toxins matrine and azadirachtin. Based on the resistance monitoring data, rotation of insecticides with different modes of action may be needed to delay or prevent the development of resistance.
